# A study protocol for a randomised crossover study evaluating the effect of diets differing in carbohydrate quality on ileal content and appetite regulation in healthy humans

**DOI:** 10.12688/f1000research.17870.2

**Published:** 2019-10-22

**Authors:** Claire S. Byrne, Dominic Blunt, James Burn, Edward Chambers, Aygul Dagbasi, Georgia Franco Becker, Glenn Gibson, Lilian Mendoza, Kevin Murphy, Carlos Poveda, Anya Ramgulam, Martina Tashkova, Gemma Walton, Chaiwat Washirasaksiri, Gary Frost

**Affiliations:** 1 Section for Nutrition Research, Department of Medicine, Imperial College London, London, UK; 2 Department of Imaging, Charing Cross Hospital, Imperial NHS Trust, London, UK; 3 Department of Food and Nutritional Sciences, University of Reading, Reading, UK; 4 Section of Endocrinology and Investigative Medicine, Department of Medicine, Imperial College London, London, UK

**Keywords:** Dietary Fibre, Carbohydrate, Gastrointestinal tract, Ileum, Colon, Gut microbiota, Nasoenteric

## Abstract

**Introduction:** A major component of the digesta reaching the colon from the distal ileum is carbohydrate. This carbohydrate is subject to microbial fermentation and can radically change bacterial populations in the colon and the metabolites they produce, particularly short-chain fatty acids (SCFA). However, very little is currently known about the forms and levels of carbohydrate in the ileum and the composition of the ileal microbiota in humans. Most of our current understanding of carbohydrate that is not absorbed by the small intestine comes from ileostomy models, which may not reflect the physiology of an intact gastrointestinal tract.

**Methods:** We will investigate how ileal content changes depending on diet using a randomised crossover study in healthy humans. Participants will be inpatients at the research facility for three separate 4-day visits. During each visit, participants will consume one of three diets, which differ in carbohydrate quality: 1) low-fibre refined diet; 2) high-fibre diet with intact cellular structures; 3) high-fibre diet where the cellular structures have been disrupted (e.g. milling, blending). On day 1, a nasoenteric tube will be placed into the distal ileum and its position confirmed under fluoroscopy. Ileal samples will be collected via the nasoenteric tube and metabolically profiled, which will determine the amount and type of carbohydrate present, and the composition of the ileal microbiota will be measured. Blood samples will be collected to assess circulating hormones and metabolites. Stool samples will be collected to assess faecal microbiota composition. Subjective appetite measures will be collected using visual analogue scales. Breath hydrogen will be measured in real-time as a marker of intestinal fermentation. Finally, an *in vitro* continuous fermentation model will be inoculated with ileal fluid in order to understand the shift in microbial composition and SCFA produced in the colon following the different diets.

**Registration:** ISRCTN11327221.

## Introduction

### Obesity

Obesity is a chronic health problem that has reached epidemic proportions globally ^1^. Obesity increases the risk of developing a range of non-communicable diseases including type 2 diabetes, cardiovascular disease, certain cancers and osteoarthritis ^2^. It has been projected that obesity rates could double by 2050, which would add £5.5 billion to the total annual expenses of the National Health Service (NHS) ^3^. The Foresight report highlighted appetite regulation as a major target in the dietary treatment of obesity ^3^. However, it is not yet fully understood how the gastrointestinal (GI) tract senses dietary content in order to suppress subsequent food intake. This knowledge could inform the development of foods or dietary regimes that increase fullness and prevent weight gain ^4^.

### The gastrointestinal tract

The GI tract is the largest endocrine organ in the body and is responsible for digesting and absorbing dietary components. The GI tract is also the host to a microbiota, which ferment undigested material. The GI tract senses changes in the luminal nutrient content and modulates neuronal and hormonal signals from the GI tract in order to help regulate appetite and food intake ^5^. The anorexigenic gut hormones peptide YY (PYY) and glucagon-like peptide-1 (GLP-1), released from enteroendocrine cells (EEC) ^5^, are examples of such GI signals secreted following a meal in a two-phase process. The first phase is thought to be mediated primarily by neural mechanisms and may help drive satiation (the sum of processes that cause meal termination). The second phase is thought to be mediated by direct nutrient sensing in the lower parts of the GI tract and to be an important long-term satiety signal. The peripheral administration of PYY or GLP-1 has previously been shown to reduce food consumption in animal models and humans, highlighting the importance of these gut hormones ^6– 10^. It has therefore been suggested that the incorporation into the diet of foods that stimulate a greater gut hormone secretion could suppress appetite to a greater extent and therefore help control body weight in the long term.

### Dietary fibre

There is an increasing amount of evidence to suggest that consumption of dietary fibre is beneficial to human health. Dietary fibre refers to carbohydrates that cannot be digested by mammalian enzymes, and thus remain in relatively intact form when they reach the caecum, where they are subsequently available for fermentation by the gut microbiota. Epidemiological evidence suggests that diets high in non-digestible fibres are associated with lower body weight gain in humans ^11, 12^. In addition, fermentable fibres have been shown to protect against weight gain and fat mass development in rodents fed a high-fat diet ^13^. Dietary fibre is thought to aid in weight management through a number of mechanisms including the promotion of satiation, increased GI transit time and stimulation of gut hormone secretion ^14^. Short-chain fatty acids, products of microbial fermentation, have also been shown to stimulate the secretion of GLP-1 and PYY ^15^. Together, this evidence suggests that fibre may be beneficial in the management of obesity.

### Importance of the cellular structure of foods

The cellular structure of foods can also be an important determinant of the subsequent impact of a food on appetite. In order for macronutrients within foods to be digested, they need to be in contact with digestive enzymes of the GI tract. In plant tissues, cell wall rupture can lead to a release of macronutrients into the extracellular environment, or enzymes can diffuse through a permeable cell wall in order to digest the encapsulated macronutrients ^16^. However, cell wall matrices or individual cell wall polysaccharides of plant foods can behave in a variety of ways during digestion. For example, some cell walls are highly impermeable and less susceptible to rupture, which leads to a reduction in the rate and extent of nutrient release and digestion. Thus, the cell walls of plants can behave as a physical barrier to digestion in the upper GI tract. The degree of domestic and industrial processing (e.g. milling, blending) of plant foods and ingredients also affects macronutrient bioaccessibility and digestion by modifying the structural integrity of the plant tissue. The importance of cell wall integrity has previously been highlighted in determining the effect of plant foods on physiological functions. These studies reported that structurally intact plant tissues tend to be digested to a lesser extent and at a slower rate, which attenuated the postprandial rise in glycaemia and/or lipaemia ^17– 19^, and may also trigger the release of lower concentrations of anorexigenic gut hormones within the small intestine, compared to foods in which the nutrients are bioaccessible. However, upon reaching the colon from the distal ileum, undigested plant material serves as a substrate for microbial fermentation, which leads to the production of SCFA and triggers the release of anorexigenic gut hormones. This process is thought to result in a more long-term satiety signal following the consumption of minimally processed plant-based foods.

### Study rationale

Within the GI tract, the colon contains the highest density of EEC ^20^. These cells express a large array of G protein coupled receptors (GPCRs) that sense nutrients and metabolites in the colonic lumen ^21^. This gives the colon the ability to regulate appetite and the GI tract in response to the nutrient environment in the colonic lumen. A major component of the digesta reaching the colon is carbohydrate, which is a major fuel source of the gut microbiota ^22^. The flow of carbohydrate into the colon from the distal ileum can radically change populations of bacteria in the colon and metabolites they produce, particularly SCFAs ^22^. However, very little is known about the type and amount of carbohydrate present in the ileum as well as the composition of the ileum microbiota in humans. Most of our current understanding of carbohydrate that is not absorbed by the small intestine comes from ileostomy models ^23^. These models may not reflect the physiology of an intact GI tract, as the gut alters following the surgery. In the present study we will use an ileum intubation method in healthy humans, which will allow us to gain a ‘real-time’ perspective on how the actual ileum content changes in response to different diets and the subsequent effect on measures of appetite and gut hormone secretion. We will also conduct an *in vitro* study in order to understand how ileum content affects the colonic gut microbiota and the production of SCFA in the colon, as this is important in determining how dietary carbohydrate drives colonic gut hormone release.

## Study objectives

### Hypothesis

We hypothesise that the consumption of minimally-processed high-fibre foods will result in more intact cellular structures and carbohydrate reaching the distal ileum and thus increasing SCFA production and PYY and GLP-1 release.

### Objectives

There are a number of objectives to the present study: to identify the impact of dietary carbohydrate on (1) the forms and levels of carbohydrates in, and (2) the microbiological profile of, the ileum, and to (3) to determine whether the carbohydrate content of the ileum relates to appetite responses and gut hormone release.

### Primary and secondary outcome measures

The co-primary outcome measures for this study are:

1. Metabolic profiling of ileal samples
2. Microbiological profiling of ileal samples

The secondary outcome measures include:

1. Microscopy of ileal samples for plant structures, cellular structures and starch granules
2. Metabolic and hormonal profiling of blood samples
3. Metabolic and microbiological profiling of faecal samples
4. Subjective appetite measurements
5. Breath H _2_ concentrations
6. Ileum samples for the inoculation of an *in vitro* continuous fermentation model

## Protocol

This study will be a randomised crossover study consisting of three separate 4-day inpatient study visits at the National Institute for Health Research (NIHR) Imperial Clinical Research Facility (CRF), Hammersmith Hospital, London, United Kingdom.

### Health screening

Healthy humans will be recruited using existing healthy volunteer databases (e.g. the Healthy Volunteer Panel at the NIHR Imperial CRF) and by advertisement in public buildings, on the internet and social media. Participants who express an interest in taking part in the study will be provided with the Patient Information Sheet (PIS). Following an initial telephone screening, potential participants will be invited to attend a health screening visit at the NIHR Imperial CRF in order to further assess their eligibility and provide written, informed consent to a member of the study team. The consent form and participant information sheet are available on Figshare ^24^. During health screening, the study protocol and the risks and benefits of participating will be explained in full and any questions that the participant may have will be answered. Participants will be asked questions about their medical history, anthropometric measurements will be collected, blood pressure (BP) will be measured, an echocardiogram (ECG) will be performed and a blood sample taken. The collected blood sample (1 x 20 ml) will be used to assess HbA1c, full blood count, liver function, renal function and blood lipids. A pregnancy test will be performed on women of childbearing age. Use of contraceptives will be noted. Inclusion and exclusion criteria will be assessed as described.

### Study population

Healthy humans aged between 18–65 years (inclusive) with a body mass index (BMI) of 18.5-30 kg/m ^2^ will be recruited for this study. A gender-balanced recruitment will be aimed. They must also show a willingness and ability to give written informed consent and to understand, to participate and to comply with the study requirements. Exclusion criteria includes: abnormal echocardiograph (ECG), screening blood results outside of normal reference values, weight change of ≥5kg in the preceding 2 months, current smokers, history of substance abuse and/or excess alcohol intake in the last 2 years, pregnancy, diabetes, cardiovascular disease, cancer, gastrointestinal disease, kidney disease, liver disease, pancreatitis, started new medication within the last 3 months likely to interfere with energy metabolism/appetite regulation/hormonal balance, antibiotic use within the last months, participation in a research study in the 12 week period prior to entering this study, any blood donation within the 12 week period prior to entering this study, vegan or vegetarian diets.

### Randomisation

Following the health screening, eligible participants will be randomised into the study. Randomisation will be performed by an independent internet and telephone randomisation company (sealedenvelope.com). Due to the different physical appearance of the diets, participants will not be blinded.

### Sample size

We aim to recruit 15 subjects. This is a pilot study in a new area of research and therefore a formal power calculation is not possible. However, a recent study in ileostomy patients investigating differences in the carbohydrate output in ileal effluent depending on the quality of carbohydrate consumed was able to detect significant differences between outcome measures in a similar number of subjects (n=9) ^18^.

### Dietary intervention

***Dietary calculations.*** All three diets are tailored to meet the energy requirements (TEE, total energy expenditure) of each participant. Using the participant’s weight and age at screening, Schofield equations will be used to calculate each participant’s basal metabolic rate (BMR) as shown in Table 1. This will then be multiplied by 238.85 to convert MJ/d to kcal/d and then by 1.2 to account for their low physical activity level (PAL) while being inpatients at the CRF.

**Table 1.** Formula for calculating total energy expenditure (TEE) (kcal/day) using the Schofield equations and physical activity level correction.

Gender	Age range (y)	Regression formula for BMR (MJ/d)	TEE calculation (kcal/d)
Male	18–29	0.063 x weight (kg) + 2.896	* 238.85 (To convert MJ to kcal) * 1.2 (PAL)
	30–59	0.048 x weight (kg) + 3.653	
	60–74	0.0499 x weight (kg) + 2.930	
Female	18–29	0.062 x weight (kg) + 2.036	
	30–59	0.034 x weight (kg) + 3.538	
	60–74	0.0386 x weight (kg) + 2.875	

BMR, basal metabolic rate. PAL, physical activity level. TEE, total energy expenditure.

***Dietary overview.*** During the 3 separate study visits, volunteers will be provided with diets differing in carbohydrate quality, which have been designed using DietPlan 6 software. In a randomised order, volunteers will receive:

- Diet 1 (low fibre (LF)-refined): This diet contains highly refined and processed carbohydrate. Foods are low in fibre and intact cell structures.
- Diet 2 (high fibre (HF)-whole): This diet is high in fibre providing a high intake of intact cellular structures. Foods have resistant cell structures such as beans, nuts, fruit and vegetables.
- Diet 3 (HF-disrupted): This diet is also high in fibre but with disrupted cellular structures. It is the same as the HF-whole diet, but foods will be milled or blended to disrupt the cellular structure.

All the diets have a similar macronutrient content (55% energy from carbohydrate, 30% energy from fat, 15% energy from protein). However, the quality of carbohydrate present is significantly different. For example, the HF-whole and HF-disrupted diets contain 46.5 g and 47.7 g dietary fibre/2000 kcal, respectively, while the LF-refined diet contains only 16.3 g dietary fibre/2000 kcal. Diets will also be analysed for their dietary fibre profiles.

Breakfast, lunch and dinner will each provide 30% of the participant’s prescribed caloric intake and an evening snack will provide the final 10%. The dietary plan for each diet is outlined in Table 2. All food provided to the participants will be prepared by the study team in the CRF Diet Kitchen according to food hygiene/food safety standards.

**Table 2.** Dietary plan for intervention diets.

Meal	Time	LF-refined	HF-whole	HF-disrupted	% E provided
**Breakfast**	9 am	Cornflakes cereal	Jumbo oats	Finely milled oats, blended	30
		Whole milk	Whole milk	Whole milk	
		White bread, toasted	German style rye bread	Rye bread made with wholemeal flour	
		Margarine	Margarine	Margarine	
		Full fat natural yoghurt	Full fat natural yoghurt	Full fat natural yoghurt	
			Oranges	Oranges, blended	
**Lunch**	1 pm	Beef lasagne	Carrot and butterbean soup (tinned)	Carrot and butterbean soup (tinned), blended	30
		Coca Cola	German style rye bread	Rye bread made with wholemeal flour	
			Margarine	Margarine	
			Apples	Apples, blended	
			Plain peanuts	Plain peanut butter	
**Dinner**	5 pm	Shepherd’s pie	Chicken breast (pre-packed)	Chicken breast (pre-packed)	30
		Coca Cola	Boiled new potatoes	Boiled new potatoes, blended	
		Wine gum sweets	Raw carrot	Boiled carrot, blended	
			Peas (tinned)	Peas (tinned), blended	
			Beetroot (pre-packed)	Beetroot (pre-packed), blended	
			Cherry tomatoes	Cherry tomatoes, blended	
			Chickpeas * (tinned) with lemon juice, olive oil, parsley, mint, black pepper, salt	Chickpea humus * (Chickpeas (tinned) with lemon juice, olive oil, parsley, mint, black pepper, salt, which are blended into a humus)	
**Snack**	9 pm	White bread	German style rye bread	Rye bread made with wholemeal flour	10
		Margarine	Margarine	Margarine	
		Medium cheddar cheese	Bananas	Bananas, blended	
			Oranges	Oranges, blended	

*Chickpea and chickpea humus recipes will be energy matched. E, energy, HF, high fibre; LF, low fibre.

***Diet preparation.*** For the LF-refined diet, breakfast will be prepared the night before serving but the bread will be toasted and the milk will be added to the cornflakes immediately before serving. The meals served at 13:00 and 17:00 will be ready meals, which will be cooked in the microwave according to the manufacturer’s instructions and the required portion will be weighed out before serving.

For the HF-whole diet, the breakfast will be prepared the day before serving and the oats will be soaked in milk from approximately 16:30 the evening before. The lunch will be prepared in the hour before serving; the apples will be cut into chunks that are easier to eat and the soup will be weighed out and microwaved just before serving. The dinner will be prepared in the hour before serving. The potatoes will be boiled for 20 min and the peas microwaved according to the manufacturer’s instructions. Both will be drained and weighed after cooking. All other foods will be served cold. The carrot, beetroot and tomatoes will be cut up into smaller pieces that are easier to eat. The evening snack will be prepared at dinner-time.

For the HF-disrupted diet, the oats will be blended in advance into a flour-like consistency. The rye bread and humous will be made in advance in-house, frozen and defrosted as needed. The breakfast will be prepared the day before serving. The oranges will be blended to a smoothie. The oats will be soaked in milk from approximately 16:30 the evening before and heated in the microwave immediately before serving. The lunch will be prepared in the hour before serving. The apples will be blended to a puree. The can of soup will be blended, the required portion will be weighed out and microwaved just before serving. The dinner will be prepared in the hour before serving. The beetroot and tomatoes will be blended to a smoothie. The potatoes and carrots will be boiled for 20 min and the peas will be microwaved according to the manufacturer’s instructions. The vegetables will then be drained, blended and weighed. The potato, carrot and peas will then be re-heated in the microwave to comply with food safety standards. All other foods will be served cold. The snack is prepared at dinner-time and the bananas and oranges will be blended.

All foods that are heated or cooked by the study team will be temperature probed before serving.

### Study visit protocol

**Figure 1.**
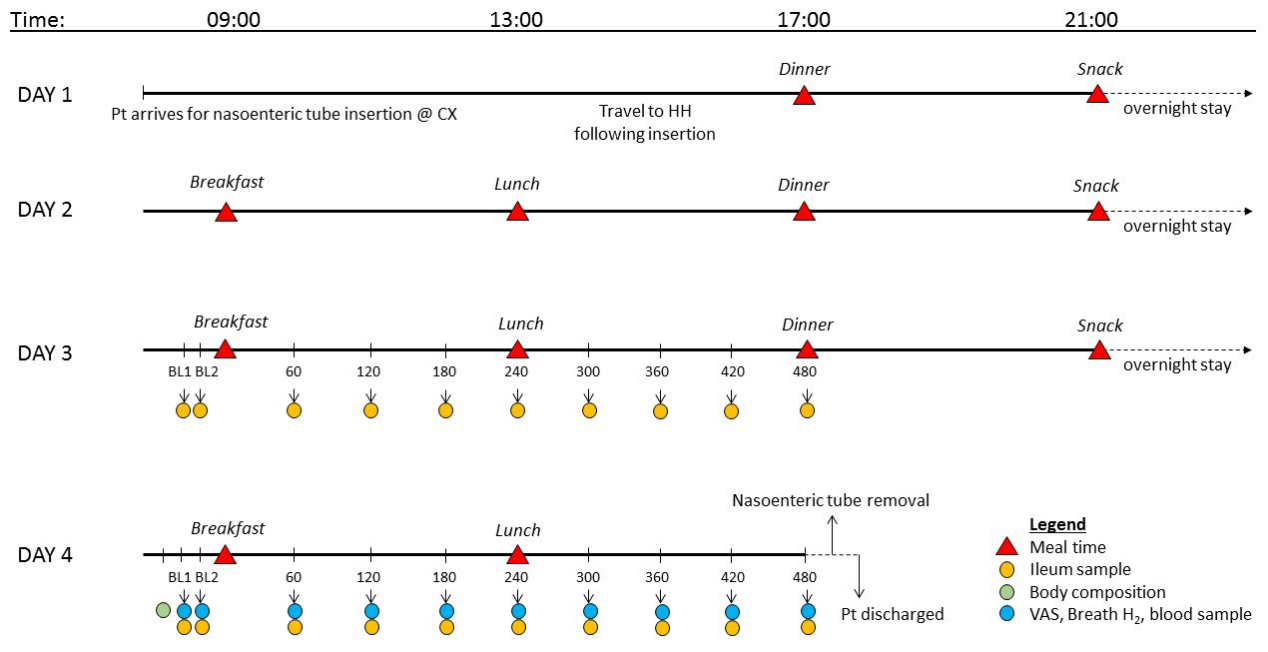
Study visit overview. Abbreviations: BL, baseline; CX, Charing Cross Hospital; HH, Hammersmith Hospital; Pt, patient; VAS, visual analogue scale.

An overview of the study visit protocol is outlined in Figure 1. Participants will attend their first study visit within 12 weeks of their screening visit. There will be a minimum wash out period of 7 days between study visits.

**Figure 2.**
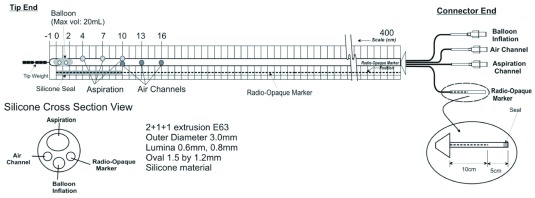
Nasoenteric tube design. Published with permission from MUI Scientific.

***Day 1: Nasoenteric tube insertion.*** Participants will be asked to arrive at the Imaging Department in Charing Cross Hospital, London, having fasted overnight and having avoided intense exercise, caffeine and alcohol the day before their study visit. Females of child-bearing age will be asked to provide a urine specimen in order to perform a pregnancy test. A custom-made nasoenteric tube ( Figure 2) will then inserted through the nose. Once it has passed the stomach and the first part of the small intestine, the balloon at the terminal end will be inflated to approximately 6 ml and will be used to carry the tube through the small intestine by peristalsis. The tube position will be confirmed by fluoroscopy throughout the tube insertion process. Dilute barium sulphate may also be administered in order to help confirm the positioning. Once the tube has reached the terminal ileum, the balloon will be deflated and the tube will be restrained from additional movement for the rest of the 4-day visit. Participants will be provided with food and drinks during the tube insertion process. However, the food provided will not be standardised to allow for flexibility to aid tube insertion. Following the tube insertion, participants will travel back to the NIHR Imperial CRF, Hammersmith Hospital, accompanied by a trained medical professional. The final position of the tube will be recorded in the participant’s notes.

***Day 1 – Start of dietary intervention.*** Participants will be fed one of the three diets over the 4-day study period according to their randomisation. The dietary intervention will start with dinner on Day 1 and end with lunch on Day 4. Meals will be provided at set times; breakfast at 09:00, lunch at 13:00, dinner at 17:00 and an evening snack at 21:00. During their stay, participants will be asked to eat all of the food that is provided and to not eat any other food including chewing gum. Participants will have free access to water, except for during sampling periods on Day 3 and 4 where water will be limited. Participants will not be allowed to leave the CRF during their visit but will have access to WiFi and TV. Participants will be asked to collect a stool sample each time they pass stool during their inpatient stay for metabolomic and microbiological analysis.

***Day 2 – Acclimatisation day.*** Day 2 will allow participants to acclimatise to their new diet and environment. Meals will be provided at set meal times and participants will have free access to water. The only samples collected on Day 2 will be stool samples.

***Day 3 – Ileal sampling.*** On day 3, ileal samples will be collected via the nasoenteric tube for metabolomic and microbiological analysis. For the 30 minutes before breakfast, the participant’s water jug will be removed. Two baseline ileal samples will be collected >10 min apart. Breakfast will be served at 9:00 am (±10 min) with 500 ml water. The time at which each participant starts eating breakfast will be considered to be 0 min. Further ileal samples will be collected every 60 min for 480 min. Lunch will be served directly after the 240 min sample with 500 ml water. Dinner will be served at 17:00, after the 480 min sample and participants will have free access to water for the rest of the day.

***Day 4 – Ileal and blood sampling.*** On the morning of day 4, participants will have an intravenous cannula inserted to allow for blood sampling and body composition will be assessed by bio-impedance analysis. For the 30 minutes before breakfast, the participant’s water jug will be removed, as on day 3. Two baseline samples will be collected >10 min apart before breakfast. At each timepoint, subjective feelings of appetite (‘how hungry/full do you feel?’) and mood (‘how nauseous do you feel?’) will be collected using a series of 100 mm visual analogue scales (VAS). The left extremity of the VAS is labelled with ‘not at all’ and the right-hand extremity is labelled with ‘extremely’. Participants will be asked to draw a vertical line on the VAS depending on how intensely they are experiencing each feeling. Baseline breath H _2 _measurements will be collected in real-time using a handheld monitor (Gastro+ Gastrolyser Breath Hydrogen Monitor, Bedfont Scientific) and will be used as a marker of intestinal fermentation ^25^. Baseline blood samples will be collected to assess blood hormones and metabolites and ileal samples will be collected via the nasoenteric tube as on Day 3. Ileal samples will be collected across both Day 3 and 4 in order to gain a full understanding of the dynamic change of the ileum environment over the study period. Once both baseline samples have been collected, breakfast will be served at 9:00 am (±10 min) with 500 ml water. The time at which the participant starts eating breakfast will be considered to be 0 min. Further samples will be collected every 60 min for 480 min. Lunch will be served directly after the 240 min sample with 500 ml water. Following the final sample at 480 min, the cannula and nasoenteric tube will be removed and participants will be discharged. **

### Sample preparation

***Stool samples.*** Stool samples will be frozen at -80°C immediately following collection and the time of collection will be recorded.

***Ileal samples.*** In order to collect ileal samples, the aspiration channel of the tube will first be flushed with a volume of water that is equal to the deadspace leading to the lumen. The air channel will be opened and the volume of water that the tube was flushed with will be taken back using a 50 ml syringe. Once the flush has been collected, the aspiration channel will be clamped using a medical clamp and the flush will be discarded. Up to 5 ml ileal sample will be collected at each sampling timepoint. Samples will be put on ice immediately following collection and frozen at -80°C. Following sampling, the aspiration channel of the tube will be flushed with a volume of water that is equal to the deadspace and both the aspiration and air channels will be closed until the next sample.

***Blood samples.*** A total of 10 ml blood will be collected at each time point and aliquoted into vacutainers: 1 ml blood will be added to a BD Fluoride Ethylenediaminetetraacetic acid (EDTA) Vacutainer; 2 ml blood will be added to a BD Lithium Heparin Vacutainer containing 20 μl/ml whole blood Aprotinin pancreatic protease inhibitor (Nordic Pharma UK Ltd, Reading, UK), 2ml blood will be added to a BD Lithium Heparin Vacutainer and 5 ml blood will be added to a BD Serum SST Vacutainer. Plasma tubes will be centrifuged immediately at 2,500 relative centrifugal force (RCF) for 10 min at 4°C. Serum tubes will be allowed to clot before centrifugation. Resulting plasma and serum will be separated and frozen at -80°C until analysis.

### Sample analysis

Samples will be analysed upon completion of the study. Metabolic and microbiological profiling of ileal and faecal samples will be performed using ^1^H-NMR spectroscopy and 16S sequencing, respectively. The metabolic and hormonal profiling of blood samples will be assessed using ^1^H-NMR spectroscopy, radioimmunoassays, Ultraperformance Liquid Chromatograpy coupled to Mass Spectrometry (UPLC-MS) and Gas Chromatography coupled to Mass Spectrometry (GC-MS). Subjective appetite will be assessed using visual analogue scales (VAS) and breath H _2_ will be measured in real-time using a handheld gastrolyser.

### *In-vitro* continuous fermentation model system

A one-stage continuous fermentation model system will be established using ileal samples from the human study to monitor *in vitro* the effect of carbohydrate quality on ileal microbiota composition and their subsequent metabolites. The system will be adapted from the three-stage colonic model described by Gibson *et al.* ^26^. Ileal samples will be collected via a nasoenteric tube ( Figure 2) as previously described and anaerobically transferred into hungate tubes, which will then be used to inoculate the *in vitro* system. Samples will be obtained from the system at baseline (t=0) and 24 hours after (t=24).

The diets from the human study will then be tested in the *in vitro* system. Diets will be digested by an *in vitro* simulation of upper gut digestion as per Mills *et al.* ^27^. The remaining products will be dialysed using a membrane of 100–200 Dalton cut off (Biotech CE Dialysis Tubing, Spectrum Europe, Netherlands). The products will be freeze-dried before their addition to the *in vitro* system. Samples will be obtained from the system 24 hours after the addition of the diets (t=48) to measure main bacterial groups using fluorescent *in situ* hybridisation (FISH) and for microbial produced metabolites using ^1^H-NMR spectroscopy.

## Data management and dissemination

### Data management

Participants will be given an anonymised personal study code number once randomised, which will be used throughout the study and in the analysis of data. The study codes will be kept on departmental databases. All personal data will be stored in locked filing cabinets in the Section of Investigative Medicine, Imperial College London. Only members of the Section of Investigative Medicine will have access to these. The Principal Investigator and the study team will have access to the final trial dataset.

### Statistical methods

A formal data analysis plan will be drawn up upon completion of the study. However, assuming the data is parametric and conforms to similar studies we have conducted, we would expect our outcome measures to be analysed using a variety of statistical methods. These will include parametric statistics and multivariate analysis using pattern recognition techniques such as principal component analyses (PCA) and partial least squares discriminant analysis (PLS-DA) and non-metric multidimensional scaling (NMDS) plots, using a number of statistical packages including SPSS, MATLAB and R.

### Data monitoring

As this is a pilot study, there are too few participants to have a data monitoring committee.

### Trial sponsor

Imperial College is the main research sponsor for this study.

### Dissemination of results

Once data analysis is complete, participants will receive a lay summary of the study’s findings. In addition, the results of the study will be presented at scientific meetings and conferences, and published in relevant high-impact journals. Research papers will be written by the study team. Authors will be included according to the International Committee of Medical Journal Editors (ICMJE) Recommendations. An anonymised dataset will be made available to the scientific community.

### Ethical approval

This study has been approved by the London – Bloomsbury Research Ethics Committee (REC) and Health Research Authority (HRA; REC Reference Number: 17/LO/0354). This study will be conducted in accordance with the recommendations for physicians involved in research on human subjects adopted by the 18 ^th^ World Medical Assembly, Helsinki 1964 and later revisions. Any substantial amendments made to the protocol, PIS or consent form will be submitted to the REC and HRA. Once these changes have been approved, these modifcations will be explained to participants and implemented with their consent.

Participants will have the choice during the consent process to agree to their samples being stored and used for future analyses. Samples will be kept in the Section of Investigative Medicine and will only be used for other research purposes that have been ethically approved.

### Adverse events

Any significant adverse event as assessed by the researchers will halt the study and the research ethics committee and sponsor will be informed as per standard protocol. All adverse events will be recorded and investigators will review each adverse event as it arises.

### Indemnity

Imperial College London holds negligent harm and non-negligent harm insurance policies, which apply to this study.

## Discussion

The maintenance of body weight throughout life is important for metabolic homeostasis. Over the last 15 years, both epidemiological and experimental evidence have highlighted the importance of carbohydrate fermentation within the gut to appetite regulation. However, our current investigative tools are not capable of characterising the complex signalling that mediates these effects in the gut. In the current study, we will investigate how the quality of dietary carbohydrate consumed affects the form and type of carbohydrate in the ileum. This will allow us to gain a deeper understanding of the composition of the digesta reaching the colon from the ileum following such diets. In addition, we will determine how this subsequently affects subjective appetite measures and gut hormone secretion. We will use an *in vitro* model, inoculated with ileal fluid, to provide a deeper understanding of how ileum contents, following diets differing in carbohydrate quality, affects the colonic gut microbiota and the production of SCFAs in the colon. This is important in identifying how dietary carbohydrate drives colonic gut hormone release and is critical in understanding the relationship between the colon and the maintenance of energy homeostasis. This knowledge may facilitate the development of food products designed for body weight maintenance.

### Study progress

The first study visit was completed in October 2017. Eight participants have successfully completed the study so far.

## Data availability

### Underlying data

No underlying data are associated with this article.

### Extended data

Figshare: A study protocol for a randomised crossover study evaluating the effect of diets differing in carbohydrate quality on ileal content and appetite regulation in healthy humans. https://doi.org/10.6084/m9.figshare.7752131.v1 ^24^.

This project contains the following extended data:

- Participant Information Sheet - Version 5 – 111017.doc (information sheet for research participants)
- Consent Form - Version 1 - 060217.doc (consent form given to each participant)

### Reporting guidelines

Figshare: SPIRIT checklist for “A study protocol for a randomised crossover study evaluating the effect of diets differing in carbohydrate quality on ileal content and appetite regulation in healthy humans”. https://doi.org/10.6084/m9.figshare.7752131.v1 ^24^.

Data are available under the terms of the Creative Commons Attribution 4.0 International license (CC-BY 4.0).
