# Supplementary material for: A study protocol for a randomised crossover study evaluating the effect of diets differing in carbohydrate quality on ileal content and appetite regulation in healthy humans
Source: F1000Res. 2019 Oct 22;8:258. Originally published 2019 Mar 5. [Version 2] doi: 10.12688/f1000research.17870.2 (PMC6904985; doi:10.12688/f1000research.17870.2)
Supplement: Peer review history

# Peer review: Reviewer response for version 2

**DOI:** 10.5256/f1000research.23079.r55597

**Published:** 2019-12-09

**Authors:** Harriët Schellekens, Marcel van de Wouw

**Affiliations:** 1 APC Microbiome Ireland, University College Cork, Cork, Ireland; 2 Department of Anatomy & Neuroscience, University College Cork, Cork, Ireland

No further comments, looking forward to seeing the outcomes of the study.

 All the best.

We confirm that we have read this submission and believe that we have an appropriate level of expertise to confirm that it is of an acceptable scientific standard.

# Peer review: Reviewer response for version 2

**DOI:** 10.5256/f1000research.23079.r55596

**Published:** 2019-12-03

**Authors:** Ellen E. Blaak

**Affiliations:** 1 Department of Human Biology, NUTRIM School of Nutrition and Translational Research in Metabolism, Maastricht University Medical Centre, Maastricht, The Netherlands

The authors have addressed my comments very well, I have no further comments.

I confirm that I have read this submission and believe that I have an appropriate level of expertise to confirm that it is of an acceptable scientific standard.

# Peer review: Reviewer response for version 1

**DOI:** 10.5256/f1000research.19542.r47113

**Published:** 2019-09-23

**Authors:** Harriët Schellekens, Marcel van de Wouw

**Affiliations:** 1 APC Microbiome Ireland, University College Cork, Cork, Ireland; 2 Department of Anatomy & Neuroscience, University College Cork, Cork, Ireland

The study protocol submitted by Byrne *et al.* is an exciting and well-thought out experiment, aimed at investigating the impact of diets with different carbohydrate quality (i.e. low fibre, whole high fibre and disrupted high fibre) on the gastrointestinal microbiota and appetite in humans. The repeated ileal sampling provides for a real time analysis of the response of the microbiota to these different diets.

**Minor comments:**

- Some lines of research indicate that different dietary fibres (e.g. inulin, rhamnose) differentially impact the gut microbiota and subsequent SCFA production (Baxter *et al.* 2019 ^1^; Reichardt *et al.* 2018 ^2^). This is well-controlled for when comparing the HF-whole and HF-disrupted groups, but not so much for the LF-refined group. It might be good to, if possible, analyse dietary fibre profile of the diets. This will increase the interpretability of the data and allow for a better comparison between the LF and HF groups, as LF will likely have a different dietary fibre profile.
- It is likely that the food intake patterns of participants prior to the study will influence their microbiota and therefore how their microbiota will respond to the different diets. Performing a food frequency questionnaire to assess their diet prior to the visit date might therefore provide valuable insight into how day-to-day food intake patterns can influence the microbiota and its response to these diets.Or any other measure to have an record of baseline food intake and food quality.
- Both males and females will be recruited, but its not stated if the aim is to recruit at a gender balanced level. Use of oral contraceptives and menstrual cycle should be recorded (oral contraceptives possibly excluded).
- Page 3, sentence: “Short-chain fatty acids, products of microbial fermentation, have also been shown to stimulate the secretion of GLP-1 and PYY.” This sentence requires a reference.

We confirm that we have read this submission and believe that we have an appropriate level of expertise to confirm that it is of an acceptable scientific standard, however we have significant reservations, as outlined above.

## References

[1] : Dynamics of Human Gut Microbiota and Short-Chain Fatty Acids in Response to Dietary Interventions with Three Fermentable Fibers. *mBio*.2019;10(1) : 10.1128/mBio.02566-18 e02566-18 10.1128/mBio.02566-18 PMC635599030696735

[2] : Specific substrate-driven changes in human faecal microbiota composition contrast with functional redundancy in short-chain fatty acid production. *ISME J*.12(2) : 10.1038/ismej.2017.196 610-622 10.1038/ismej.2017.196 29192904PMC5776475

# Response

**Published:** 2019-10-11

**Authors:** Aygoul Dagbasi

**Affiliations:** Imperial College London, UK

Thank you for your time to review our article and provide valuable comments.

We agree with all your comments.

We will be performing a direct analysis of the dietary fibre profile of each dietary intervention to be able to better interpret the data as suggested.  As you highlighted, high fibre groups (whole and blended) will have the same profiles since these diets consist of the same foods however the low fibre group will have a different profile.

The cross-over design of the study allows each participant to act as their own control. This will eliminate confounding factors like habitual dietary intake and gut microbiota profile. Current dietary assessment methods provide an inaccurate measure of dietary intake (Shim *et al. *2014). As a result of these; we decided not to collect dietary intake data. We will be excluding participants following extreme diets such as vegan or vegetarian diets.

We will aim to recruit at a gender-balanced level. We will note information regarding females’ contraception methods and menstrual cycles.

Thank you again for your valuable comments

# Peer review: Reviewer response for version 1

**DOI:** 10.5256/f1000research.19542.r45303

**Published:** 2019-09-05

**Authors:** Ellen E. Blaak

**Affiliations:** 1 Department of Human Biology, NUTRIM School of Nutrition and Translational Research in Metabolism, Maastricht University Medical Centre, Maastricht, The Netherlands

This is an interesting study design investigating the role of the ileum and its microbiota in the processing of dietary fibers on 3 different diet, a low fiber diet and 2 high fiber diets, one with intact cellular structures and the other one with disrupted cell structures. This study gives a real time perspective how the ileum content changes in response to different diets and is therefore unique. 

 The hypothesis may be formulated a little bit sharper, the idea is that minimal processed fiber may lead to a higher fermentation in the colon with a more beneficial metabolic profile, how exactly is this question answered since fermentation and SCFA production is not directly measured in the colon?

 Would an increased fermentation in the ileum also be beneficial?

 How much time does the sampling of the ileum take? Is also the total microbial content determined in ileal and fecal samples, expressed per what?

 What is the period between the visits i.e. the washout period?

 The in vitro approach may be an interesting addition. Sampling takes place at baseline and at 24 h, why are these time points chosen and is more frequent sampling a possibility?

 Looking at the effect of whole diets in the 'in vitro' system, diets are first freeze dried, to what extent are the cellular structures maintained in this procedure and to what extent are the outcomes still relevant for the in vivo conditions?

 Overall, a challenging and interesting study protocol.

I confirm that I have read this submission and believe that I have an appropriate level of expertise to confirm that it is of an acceptable scientific standard, however I have significant reservations, as outlined above.

# Response

**Published:** 2019-10-11

**Authors:** Aygoul Dagbasi

**Affiliations:** Imperial College London, UK

Thank you for your time to review our article and provide valuable comments.

We agree with all your comments.

We will be changing our hypothesis as per your advice to fit the study design better.

We do think that an increased fermentation in the ileum would also be beneficial in addition to the colonic fermentation. This is because GLP-1 and PYY secreting L-cells increase in number along the GI tract with the highest numbers found in distal ileum and colon (Suzuki et al., 2018). Therefore the composition of the ileal microbiota and their subsequent metabolites including SCFAs can be sensed by the dense population of ileal L-cells and potentially have a profound effect on appetite hormone secretion and host metabolism.  We are hoping to answer this question better with our study results.

We had a trial run of ileal sampling which took around 5-10 minutes per sample. The microbial content will be expressed as Log 10 numbers per ml of ileal/faecal content as measured by flow-FISH (fluorescent in-situ hybridisation) and as ratios when measured by 16s rRNA sequencing.

The washout period will be minimum of 7 days.

The in-vitro system takes 24 hours to stabilise after inoculation and after the addition of diets therefore any samples taken in between would be inaccurate. Sampling can continue after 24 hours however since the system is stabilised, no change is anticipated after this period.

Microscopy pictures were taken after the diets were freeze dried. These revealed that this method is not disruptive to the cellular structures. The slides were stained with iodine which showed carbohydrate still present in intact cellular structures for Diet 2 (Whole Diet).

Thank you again for your valuable comments.
